# Recent Advancements in Prognostic Factors of Epithelial Ovarian Carcinoma

**DOI:** 10.1155/2014/953509

**Published:** 2014-10-29

**Authors:** Mohammad Ezzati, Amer Abdullah, Ahmad Shariftabrizi, June Hou, Michael Kopf, Jennifer K. Stedman, Robert Samuelson, Shohreh Shahabi

**Affiliations:** ^1^Department of Obstetrics and Gynecology, Washington Hospital Center, 110 Irving Street NW, Washington, DC 20010, USA; ^2^Department of Obstetrics and Gynecology, The University of Texas Southwestern Medical Center, 5323 Harry Hines Boulevard, Dallas, TX 75390, USA; ^3^Department of Obstetrics, Gynecology and Reproductive Biology, Danbury Hospital, 24 Hospital Avenue, Danbury, CT 06810, USA; ^4^Department of Pathology and Laboratory Medicine, School of Medicine, Tufts University, 800 Washington Street, Boston, MA 02111, USA; ^5^Division of Gynecologic Oncology, Department of Obstetrics and Gynecology and Women's Health, Albert Einstein College of Medicine and Montefiore Medical Center, Montefiore Centennial, 3332 Rochambeau Avenue, Bronx, NY 10467-2836, USA; ^6^Department of Medicine, Danbury Hospital, 24 Hospital Avenue, Danbury, CT 06810, USA

## Abstract

Ovarian cancer remains the most common cause of gynecologic cancer-related death among women in developed countries. Nevertheless, subgroups of ovarian cancer patients experience relatively longer survival. Efforts to identify prognostic factors that characterize such patients are ongoing, with investigational areas including tumor characteristics, surgical management, inheritance patterns, immunologic factors, and genomic patterns. This review discusses various demographic, clinical, and molecular factors implicating longevity and ovarian cancer survival. Continued efforts at identifying these prognosticators may result in invaluable adjuncts to the treatment of ovarian cancer, with the ultimate goal of advancing patient care.

## 1. Introduction

There has been significant progress in the management of ovarian cancer, yet the disease still has the highest mortality of all pelvic malignancies combined. In the United States, in 2013, approximately 22,000 new cases of ovarian cancer will be diagnosed and over 14,000 women will die due to the disease [1]. Primary reasons for this poor prognosis include late stage of presentation due to the absence of symptoms, lack of effective screening tools, and the development of recurrent disease that is resistant to chemotherapy. The most common ovarian cancer is the epithelial subtype, of which over 70% of patients are presented with the advanced-stage disease, where the long term survival rate (10 years) is estimated at 15–30%; in contrast the survival rate for early stage disease exceeds 90% [2–5].

The disparity in prognosis is largely a function of disease stage, which is the most important prognostic variable for ovarian cancer survival. However, a subgroup of patients is seemingly cured after standard therapy. Studies have also concentrated on the prognostic importance of tumor characteristics, surgical conditions, and demographic factors. More recently, attention has focused on the significance of molecular features, which are considered a reflection of tumor biology and might thus be utilized to predict response to various therapeutic modalities and determine the long term survival.

## 2. Age

Age is a widely accepted prognostic factor of ovarian cancer [6, 7]. Generally, a younger age at diagnosis portends a better prognosis, with women younger than 65 years having at least 2 years longer median survival compared to women older than 65 years; older women also have an increased risk of recurrence and death [8, 9]. Age remains an independent prognostic factor after controlling for common confounding factors, such as performance status and medical comorbidities. Younger-aged women tend to have less invasive and well differentiated cancers and fewer comorbidities compared to older counterparts, yielding a more favorable overall prognosis [10, 11].

## 3. Performance Status

The Gynecologic Oncology Group adopted the Eastern Cooperative Oncology Group/World Health Organization scoring criteria for quantitative assessment of a patient's general health at the time of cancer diagnosis and prior to the start of treatment. Based on this system, the following performance status scores are given [12]: 0 is asymptomatic; 1 is symptomatic but completely ambulatory; 2 is symptomatic, <50% in bed during the day; 3 is symptomatic, >50% in bed, but not bedbound; 4 is completely bedbound.

A better performance status generally confers a greater tolerance to various therapeutic modalities, from surgery to chemotherapy, and perhaps motivates the adoption of a more aggressive treatment plan by clinicians. Several studies support this rationale and confirm the independent prognostic significance of performance status [9, 13–15].

## 4. Tumor Stage and Ascites

Staging systems describe the severity of a patient's cancer in a terminology that is shared across medical disciplines. While staging was originally developed to optimize treatment planning, a lower stage at diagnosis generally translates into a superior clinical outcome and improved survival [5, 16]. The International Federation of Gynecology and Obstetrics (FIGO) developed the ovarian cancer staging system. This system emphasizes surgical evidence and correlates with the widely used TNM classification system. The FIGO system has been extensively studied over the past two decades and its independent prognostic significance is well established [13, 17–19]. Patients with stage I disease largely enjoy a greater than 95% 5-year survival, versus a less than 10% 5-year survival of patients with stage IV disease [20–22].

From a clinical perspective, a higher stage signifies more extensive disease that is less likely to be optimally debulked, compared to a tumor that is confined to the pelvis. The radical surgeries needed to achieve optimal surgical debulking in extra-pelvic (stage III) and extra-abdominal (stage IV) disease also correlate with a higher incidence of perioperative morbidities. Additionally, the presence of a malignant pleural effusion, in the absence of any other criteria for stage IV disease, indicates a significantly poorer prognosis compared to stage III disease [23]; this finding further emphasizes the prognostic significance of the FIGO staging system. A biological explanation for this significance may lie in the possibility that a higher stage reflects a change in tumor biology, to a more aggressive state [23].

Ascites distinguishes a poorer prognosis in early-stage disease, contributes to the morbidity associated with advanced disease and is thus an important component of the FIGO staging system. Stage I and II diseases are upstaged to subclass C when there are malignant cells detected in ascitic fluid. In terms of management, patients with early-stage disease and ascites cannot be surgically cured. Ascites is thus considered a marker of unfavorable prognosis in early-stage disease and studies suggest that these patients should receive adjuvant chemotherapy following surgical debulking. However, optimal treatment for early-stage disease remains to be determined [24–27]. Considering the aforementioned prognostic significance of the FIGO staging system, it follows that several studies report an independent prognostic significance for the presence or absence of ascites in advanced-stage disease [17, 28–30]. The significance of ascites in advanced-stage disease is further recognized by its incorporation into a nomogram validated to predict survival in epithelial ovarian cancer [31, 32].

## 5. Tumor Grade

The degree of cellular differentiation, or tumor grade, has long been considered a factor influencing tumor behavior and affecting survival. This understanding derives from the fact that poorly differentiated cells represent a higher degree of aberrant mitoses, which equates with a more aggressive cancer cell behavior. However, studies conflict over the association between tumor grade and clinical outcome [9, 11, 13, 17, 29, 33, 34]. Part of the controversy results from the absence of a universally accepted grading system [35, 36]. A majority of pathologists employ a system introduced by FIGO in the 1970s that uses cellular architecture as the defining characteristic of tumor grade [37]. A subsequently developed system incorporates cellular architecture, nuclear grade, and mitotic activity [38, 39]; this system correlates more precisely with lymph node metastasis and survival. It seems that histologic subtype (see below) outperforms the tumor grade in prediction of survival, especially when combined with molecular markers [40].

## 6. Histopathologic Subtype

A paradigm shift has recently occurred in the classification of ovarian cancer. Based on morphologic and molecular features, epithelial ovarian cancer is recognized as a diverse group of tumors, amenable to a dualistic classification model. Type I tumors include low-grade serous, low-grade endometrioid, clear-cell, mucinous, and transitional cancers. Type II tumors include high-grade serous, undifferentiated, and malignant mixed mesodermal cancers. Type I tumors generally demonstrate slower growth and a lower stage at diagnosis; these tumors also tend to lack mutations involving TP53 and are generally not sensitive to platinum-based chemotherapy. Cell types reflect differences in underlying molecular profile and thus in sensitivity to current chemotherapy. Type II tumors display an aggressive nature and are usually advanced in stage at diagnosis; the majority of these tumors harbor TP53 mutations and are initially sensitive to platinum-based chemotherapy. Platinum sensitivity is pivotal to the treatment of advanced ovarian cancer, for which platinum-taxane combination chemotherapy remains the standard of care following surgical debulking. Tumor-cell type is shown to be the most relevant histologic prognostic factor in advanced ovarian cancer treated with platinum/paclitaxel [40].

Recent studies indicate that low-grade serous carcinoma has a significantly better prognosis than high-grade serous carcinoma [41, 42]. In advanced epithelial ovarian cancer, recent meta-analyses report that the mucinous and clear-cell subtypes are associated with worse outcome [9, 14, 29, 43, 44]. Challenges to determining the prognostic importance of tumor histology include misclassification in studies that lack centralized histopathological review, heterogeneity of study populations, and small sample sizes.

## 7. Obesity

An extensive United States population-based study demonstrated an association between increased body mass index (BMI) and increased risk of death from all cancers. This study further reported a direct relationship between increasing BMI and increasing risk of mortality from ovarian cancer; women with a BMI of 35 and above had a relative risk of death of 1.51 compared to women with a normal BMI [34]. However, the authors noted that several studies have not shown such an association and the literature is inconsistent with regard to the etiologic relationship between obesity and ovarian cancer and the effect of obesity on survival [45–54]. Recently, a link has been found between the postmenopausal hormonal therapy and the effect of BMI on ovarian cancer prognosis; and given the role of the leptin in progression of various cancers, a hormonal mechanism is suggested. Indeed higher levels of leptin in ovarian cancer patients correlate with worse clinical outcome [55, 56].

## 8. Surgical Debulking

In accordance with a Gompertzian model of general tumor kinetics, a greater initial surgical reduction of tumor burden results in improved 5-year survival with adjuvant chemotherapeutic regimens. This understanding is based on the concept that the growth fraction of a tumor decreases with time; hence, chemotherapy destroys tumor cells based on where a tumor is on its growth curve. Smaller tumor volumes are more readily destroyed than larger volumes because the growth fraction of the smaller volume is higher and thus the fractional cell kill by a chemotherapeutic agent is also higher [57, 58]. The success of cytoreductive surgery, as reflected by the size of residual tumor, has been consistently demonstrated as one of the most influential factors of both progression-free and overall survivals [9, 13, 19, 21, 59–61]. It is noteworthy that no randomized trial has been conducted to support this understanding, and almost all supportive studies are retrospective and incorporate various adjuvant chemotherapy regimens. In advanced disease, one randomized trial demonstrates that neoadjuvant chemotherapy, wherein chemotherapy is administered before attempting cytoreductive surgery, does not improve survival [62].

A recent Cochrane Database Review evaluated the effectiveness of optimal primary cytoreductive surgery for women with advanced epithelial ovarian cancer and assessed the impact of various residual tumors sizes on overall survival [63]. Analyses showed that overall survival and progression-free survival were significantly prolonged for women who had no grossly visible residual disease following primary debulking surgery. When comparing greater than one centimeter versus less than one centimeter of residual tumor, survival estimates also favored the lower volume residual disease group. Finally, there was no significant difference in overall survival and only a borderline difference in progression-free survival when residual disease of greater than two centimeters and less than two centimeters were compared. The authors concluded that complete cytoreduction to no grossly visible disease should be the goal of primary surgery for advanced-stage epithelial ovarian cancer but noted that the retrospective nature of the data precluded a definitive understanding of whether the surgical intervention or patient-related and disease-related factors were responsible for improved survival [63]. Interestingly, a study by the Gynecologic Oncology Group found that women who presented with large volume disease and were optimally cytoreduced to less than 1 centimeter residual tumor had inferior survival compared to women who initially presented with disease of one centimeter or less, which raises the possibility of biologic factors related to disease bulk being as important as the degree of achievable surgical cytoreduction [64].

Indeed, it has recently been shown that innate tumor features determine cytoreductibility of women with ovarian carcinoma; preoperative CA-125 and P53 can determine if the tumor would be cytoreductible or not. According to a multivariate analysis, patients with tumors that strongly express p53 are significantly less likely to achieve complete cytoreduction than the patients whose tumors had mild or moderate p53 expression [65].

Surgeon subspecialization also impacts the success of initial cytoreductive surgery and survival. Patients with early and advanced-stage disease are both more likely to receive optimal cytoreduction and more likely to experience improved median and overall survivals when operated on by a gynecologic oncologist [66].

The success of neoadjuvant chemotherapy is also an important prognostic factor for patients with ovarian cancer who are not candidates for primary surgical debulking due to the extent of their disease. Unfortunately, some of these patients are refractory to initial treatment and therefore incur a poor prognosis. A recent retrospective study was published that evaluated actionable targets in patients who received neoadjuvant chemotherapy prior to surgical debulking. Of note, hepatocyte growth factor (HGF) and its receptor c-MET were enhanced in those with aggressive ovarian cancer and were associated with refractory disease [67]. The HGF/c-MET is a known growth-factor signaling pathway. The development of novel agents to target the HGF/c-MET pathway would allow for a more tailored approach for these patients with refractory disease [67].

## 9. Long-Term Prognostic Factors in Recurrent Ovarian Cancer

Prognostic factors in recurrent ovarian cancer slightly differ from the primary tumors of the same type. Several studies using multivariate analysis with age, histology, grade, and residual tumor after first-look surgery have demonstrated that residual disease after initial surgery is the main factor significantly related to survival in patients treated with second look laparotomy. Accordingly, cytoreduction at second look laparoscopy will benefit survival only if microscopic residuals remained and this effect is only seen in those patients who start with less than 1 cm of disease. Also tumor grade was associated with survival in recurrent ovarian cancer [68–72]. In recurrent ovarian cancer, patients with the nonmucinous/clear-cell type, including serous, endometrioid, and other types, have significantly more favorable outcome compared with patients with the mucinous/clear-cell histological type [73].

Additionally, several molecular studies have shown differential expression of few genes in long-term survivors. In this regard, the MAL (myelin and lymphocyte protein) gene, implicated in conferring resistance to cancer therapy, is differentially upregulated in short-term survivors (three-fold increase compared with long-term survivors and 29-fold increase compared with early-stage patients) [74]. Similarly, three other genes, namely, CYP4B1, CEPT1, and CHMP4A are differentially regulated in patients who suffer from recurrent ovarian cancer disease [75].

## 10. Hereditary Ovarian Cancer

Approximately 5% to 10% of epithelial ovarian cancers are associated with an inherited genetic predisposition, of which there are two recognized syndromes, inherited in an autosomal dominant pattern: the breast and ovarian cancer syndrome, linked to mutations in the BRCA1 and BRCA2 genes and accounting for approximately 90% of hereditary ovarian cancer cases, and the hereditary nonpolyposis colorectal cancer syndrome, accounting for approximately 5% of hereditary ovarian cancer cases [76, 77]. The BRCA genes do not demonstrate equal penetrance and the lifetime risk of developing epithelial ovarian cancer for women with a BRCA1 or BRCA2 mutation is up to 66% and 27%, respectively. Notably, women of Ashkenazi Jewish decent have an approximately 2% carrier rate of a BRCA germ line mutation, and a BRCA mutation is found in up to 62% of ovarian cancers in this population [78, 79]. Although attempts to identify the effects of BRCA mutations on ovarian cancer survival have yielded mixed results, most studies demonstrate a survival benefit for ovarian cancer patients with BRCA mutations compared to their sporadic counterparts [80, 81]. The frequency of poorly differentiated carcinoma is higher in BRCA related ovarian cancer compared to sporadic cases [80–83]; BRCA mutations have also been associated with a higher rate of cellular proliferation [84]. Poorer differentiation due to accelerated proliferation seems to convey a worse prognosis. Indeed reduced expression of BRCA1 is a common occurrence in advanced ovarian cancer [85].

Several mechanisms can account for this seemingly paradoxical observation that harboring BRCA1 mutations leads to a better prognosis. Ovarian cancers associated with BRCA mutations, particularly BRCA1, present at a younger age compared to sporadic cases [80, 81]. While younger age at diagnosis is a well-established favorable prognostic factor, BRCA carrier status remains an independently significant prognostic factor after adjusting for the effects of age. Studies have established that the BRCA1 and BRCA2 genes are significantly involved in DNA damage recognition and homologous repair (HR) [86, 87]. Higher rate of cell proliferation and poor differentiation, in conjunction with impaired HR due to mutated BRCA genes, may contribute to an improved response to the DNA-damaging effects of platinum-based chemotherapy and probably a better prognosis [88–91]. This effect is similar to the setting of impaired HR induced by poly(ADP-ribose) polymerase (PARP) inhibitors which enhance the cytotoxic effect of DNA-damaging chemotherapy [92, 93].

## 11. Immunological Factors

In the case of ovarian cancer, the protective role of the immune system has been demonstrated as a survival benefit conferred by tumor infiltrating lymphocytes [94, 95]. This benefit is more pronounced among patients with higher CD8^+^ cell counts, as well as those with higher CD8^+^ to CD4^+^ cell ratios [95, 96]. Notably, only T-cell infiltration in tumor islets demonstrates a survival benefit, while the presence of T-cells in stroma does not suggest a benefit. Additionally, the presence of CD4^+^ CD25^+^ T-cells, known as regulatory T-cells—a subset of T-cells with potent immunosuppressor activity, may predict poor clinical outcome [95, 96].

Likewise, overexpression of certain human leukocyte antigens (HLAs) and underexpression of other HLAs have been implicated in the interaction of the host immune response and ovarian cancer cells. Overexpression of human leukocyte antigen-G (HLA-G) may serve as one of the mechanisms by which ovarian cancer cells evade immune surveillance [97, 98]. HLA-G is a nonclassical major histocompatibility complex class I molecule, and there is substantial experimental evidence to support its role in suppressing the immune response. On the other hand, underexpression of HLA class I molecules results in diminished recognition of tumor cells by cytotoxic CD8^+^ T-cells. Although this downregulation of HLA class I has been correlated with higher tumor stage [99], there is inconsistent evidence that it has independent prognostic significance in ovarian cancer [99, 100].

## 12. Molecular Profiling

The most frequently studied putative molecular biological prognostic factors in ovarian cancer are the tumor suppressor protein 53 (p53), the oncogenes epidermal growth factor receptor (EGFR) and human epidermal growth factor receptor 2 (HER-2/neu). Results of a recent meta-analysis show that p53, EGFR, and HER-2/neu immunostainings do not have a strong direct relationship with survival, although likely their respective pathways do influence patient prognosis [101].

Additional biomarkers have also been previously identified as potential targets to identify prognosis and appropriate adjuvant chemotherapy after surgical debulking; however, the results have been inconclusive. It seems that, due to these conflicting results, meta-analysis studies are very helpful. For example, a recent meta-analysis evaluated Bcl-2, EGFR, GST, LRP, p16, p21, P-pg, and TNF-*α* and their relation to patient survival and response to treatment. Both EGFR and P-pg enhancements were prognostic factors for poor overall survival (OS) and poor progression free survival (PFS). Of additional utility, LRP, P-pg, and TNF were shown to be possible targets that are associated with response to platinum-based chemotherapy [102].  Table 1 shows a summary of predictive and prognostic protein biomarkers implicated in epithelial ovarian cancer [103].

The application of novel approaches based on genomic and proteomic studies has made it possible to obtain a vast amount of data about the molecular platform on which tumors arise and grow. A better understanding of these mechanisms could prove to be invaluable in sketching a roadmap towards the individualization of cancer treatment, with the ultimate goal of developing the least toxic treatment regimens to improve survival. Recently, The Cancer Genome Atlas (TCGA) project completed the first comprehensive genomic analysis of high grade serous ovarian cancer (HGS-OvCa), which accounts for the most ovarian cancer patient deaths. Using approximately 1500 intrinsically variable genes, The Cancer Genome Atlas Research Network found four clusters using unsupervised nonnegative matrix factorization that were termed “Differentiated,” “Immunoreactive,” “Mesechymal,” and “Proliferative.” These four clusters were not different regarding survival [104].

Nevertheless, in accordance with previous studies, TCGA found that almost all HGS-OvCa tumors are characterized by mutations in the tumor suppressor gene TP53 [105–107]. Interestingly, a study derived from TCGA discovered that HGS-OvCa patients without mutated TP53 are more chemoresistant and have poorer survival compared to patients with mutated TP53 [108]. TCGA additionally found HGS-OvCa displays statistically recurrent somatic mutations in nine genes, with over 20% of HGS-OvCa tumors harboring a BRCA1 or BRCA2 mutation. As previously discussed, defective homologous recombination (HR) due to BRCA mutations may render cancer cells more sensitive to PARP inhibitors. TCGA discovered genomic alterations involving other HR genes and estimated that approximately half of all HGS-OvCa tumors may harbor HR defects, which could have implications for trials of PARP inhibitors. The project also identified twenty-two amplified, overexpressed genes that are potential targets for therapeutic inhibitors. Another recent study integrated TCGA data and validated three genes (CYP4B1, CEPT1, and CHMP4A) in HGS-OvCa from patients likely to be cured by initial cytoreductive surgery and adjuvant chemotherapy; the precise role of these genes in ovarian cancer pathophysiology remains to be elucidated [109]. While the mutational spectrum delineated by TCGA is distinct for HGS-OvCa, the findings indicate that subtype-stratified care and targeted therapies may lead to future improvements in ovarian cancer treatment. In a similar approach by OVCAD consortium, by applying a gene signature comprised of 112 genes, it was shown that, in advanced-stage serous ovarian cancer, two approximately equally large molecular subtypes exist, independent of classical clinocopathological parameters and presented with highly different whole-genome expression profiles and a markedly different overall survival [110]. By making the transition from the gene as the unit of phenotypic affiliation to the molecular network as the unit of analysis, both the survival can be better predicted and also the treatment can be better stratified. Thus, instead of using individual genes, the network unit can be considered as a biomarker; for example, PDGF network (18 genes) is shown in several studies to outperform the 193-gene signature in the TCGA database [111].

Furthermore, the development of novel agents based on actionable targets is dependent on their reproducibility. It has been reported that pathways and pathway-based genes are more reliable biomarkers compared to single gene markers as they are more stable and are better at identifying the subtype of cancer across data sets [112]. Additionally, utilizing multidimensional biomarkers has greater predictive value for aggressive disease and therefore greater clinical application than employing single dimension evaluation [113]. One such pathway that has been identified in epithelial cancers, including ovarian, is the BMI1 pathway, which is associated with pluripotent potential of a cell. This has been shown to reduce response to chemotherapy, decrease time to disease progression, and increase the risk for metastatic disease. The ability to target this pathway and individualize treatment for individuals with this biomarker could greatly improve survival [114].

As a sight for the future, in order to standardize diagnosis and prognosis regimens, we see that there is a strong relation between future improvements and canonical biomarkers. Using well-recognized quantitative features to encode cancer and other phenotypes based on canonical biomarkers will connect different laboratories and give such ability to record marker-based data interchangeably among them. As a result, this relation, outcomes, and information will incorporate genomic cancer information as a matter of course, augmenting current cancer classification protocols and specifying the treatments protocols [112].

This review covered various demographic, clinical, and molecular factors that influence the survival of patients with ovarian cancer. There is a concerted effort to better understand the basis of factors implicating the longevity with this disease at the clinical and molecular levels. Currently known factors affecting patient's outcome include age, performance status, and histopathologic subtype; however, new information regarding the patient's biomarkers is likely to generate the greatest predictor of patient outcome. Therefore, continued efforts at identifying prognosticators of survival may result in invaluable adjuncts to the treatment of ovarian cancer, with the ultimate goal of advancing patient care and survival.

## Figures and Tables

**Table 1:** A summary of predictive and prognostic protein biomarkers in epithelial ovarian cancer.

Oncogenes and tumor suppressors	Tumor suppressor p53
	WT1

Proliferation markers	Ki67
	Proliferation cell nuclear antigen or PCNA
	Topoisomerases: topo II

Cell cycle regulators	Cyclins (E, D1, D3, and A)
	Cyclin inhibitors: p21, p27, p57, and p16

Apoptosis	Extrinsic apoptotic pathway
	TRAIL and receptors
	TRAIL-R1/DR-4 and TRAIL-R2/DR-5
	TRAIL-R3/DcR1, TRAIL-R4/Dcr2, and TRAIL-R5
	Fas and Fas-L
	Intrinsic apoptotic pathway
	The Bcl-2 family members (Bax and Bcl-2)
	Caspases
	Initiator: caspase-8
	Effector: caspase-3/CPP32
	Inhibitor of apoptosis (IAPs) including Survivin and X-linked IAP (XIAP)

DNA repair enzymes: BRCA1 and BRCA2, PARP-1, and ERCC1	

Markers of angiogenesis	Markers of microvascular density (CD31 and CD34)
	Markers of proteins involved in angiogenesis (VEGF, HIF, COX-2, and MMPs especially MMP-9 and MMP-7)

Immunological factors	T-cells, inhibitory T-Regs cells, cytokines, and costimulatory or inhibitory molecules expressed by immune cells or tumor cells

Tyrosine kinase receptors (TKR)	Epidermal growth factor receptor (EGFR), Her-2, ErbB3, and ErbB4
	Ephrin B receptors and other TKR
	EphB4
	Hepatocyte growth factor receptor (Hgf/Met) and its ligand, c-Met
	The signaling pathway of tyrosine kinase receptors: Erk, PI3K, Akt, and NF-*κ*BI

Epithelial-cadherin/beta-catenin	
